# Measuring replication competent HIV-1: advances and challenges in defining the latent reservoir

**DOI:** 10.1186/s12977-018-0404-7

**Published:** 2018-02-13

**Authors:** Zheng Wang, Francesco R. Simonetti, Robert F. Siliciano, Gregory M. Laird

**Affiliations:** 1 Department of Pharmacology and Molecular Sciences, Johns Hopkins University School of Medicine, Baltimore, MD 21205 USA; 2 Department of Medicine, Johns Hopkins University School of Medicine, Room 879, Edward D. Miller Research Building, 733 N. Broadway, Baltimore, MD 21205 USA; 3 Howard Hughes Medical Institute, Johns Hopkins University School of Medicine, Baltimore, MD 21205 USA

**Keywords:** HIV-1 persistence, Replication-competent HIV-1, Measuring HIV-1 latent reservoir

## Abstract

Antiretroviral therapy cannot cure HIV-1 infection due to the persistence of a small number of latently infected cells harboring replication-competent proviruses. Measuring persistent HIV-1 is challenging, as it consists of a mosaic population of defective and intact proviruses that can shift from a state of latency to active HIV-1 transcription. Due to this complexity, most of the current assays detect multiple categories of persistent HIV-1, leading to an overestimate of the true size of the latent reservoir. Here, we review the development of the viral outgrowth assay, the gold-standard quantification of replication-competent proviruses, and discuss the insights provided by full-length HIV-1 genome sequencing methods, which allowed us to unravel the composition of the proviral landscape. In this review, we provide a dissection of what defines HIV-1 persistence and we examine the unmet needs to measure the efficacy of interventions aimed at eliminating the HIV-1 reservoir.

## Defining the latent reservoir for HIV-1

Despite effective antiretroviral therapy (ART), HIV-1 persists in all infected individuals as proviral DNA integrated within long-lived memory CD4^+^ T cells [1, 2]. Early studies of infected donors on suppressive ART demonstrated that a subset of these quiescent proviruses could be induced to replicate in tissue culture, comprising what we now consider the latent reservoir [3–8]. However, subsequent investigation has revealed that a complex population of both replication-competent and defective proviruses exists within every infected individual [9–17]. This population of persistent proviruses not only archives the cumulative viral genotypic diversity, which arose over the course of untreated infection, but also serves as the primary barrier to curing HIV-1 infection. Recent studies are changing our understanding of what defines the HIV-1 latent reservoir, how we quantify it, and how we leverage these quantitative data to predict the dynamics of viral rebound (or lack thereof) in infected individuals who interrupt suppressive ART.

**Fig. 1:**
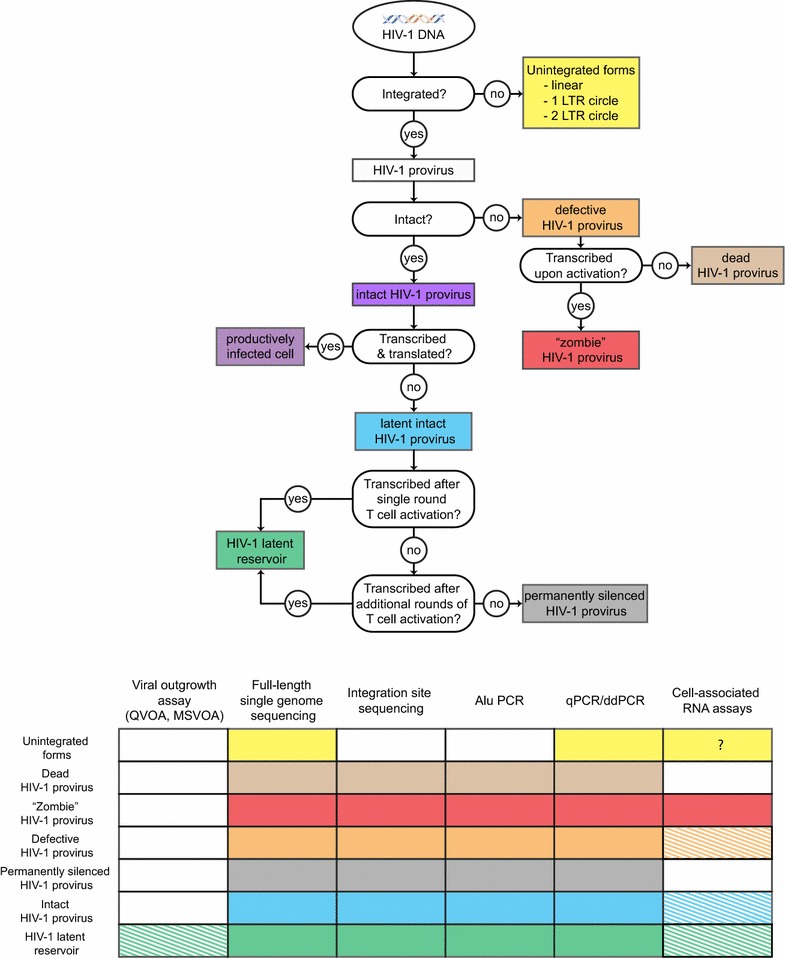
Classification of HIV-1 DNA present in HIV-1 + individuals on suppressive ART. A decision tree describing the classification of HIV-1 DNA based on integration, genomic integrity/replication competence, transcription state, and ability to be induced by T cell activation. A complementary table is provided indicating which forms of persistent HIV-1 DNA are detected by common assays in the field. Hatched patterns indicate that the relevant assay detects only a subset of viruses in a given category

Given the complexity of HIV-1 persistence, it is critical that we employ accurate and precise definitions in our discussion. ***Persistent HIV*-*1*** is an all-encompassing term that includes all forms of integrated proviral DNA that persist in infected individuals on the time-scale of years despite fully suppressive ART (Fig. 1). This term does not define the ability to replicate; several cross-sectional studies have revealed that a diverse landscape of intact and defective persistent proviral DNA can be found in infected individuals [10, 12, 13, 15, 16]. Furthermore, persistent proviruses can exist in three distinct states of viral gene expression. First, a subset of persistent proviruses are undergoing active gene expression, producing LTR-driven RNA transcripts. The fraction of persistent proviruses undergoing active transcription at any given time, the nature of said transcripts, and the clinical implications of their expression are under active investigation [18–26]. Second, a subset of proviruses may exist in a functionally dead state, in which LTR-driven initiation of transcription is not possible, due to major genomic defects or irreversible epigenetic silencing. Finally, persistent proviruses can exist in a latent state. ***Latency*** is best defined as a reversible state of non-productive infection. Latent proviruses are those that could theoretically be induced to express LTR-driven transcripts, but at a given time do not initiate or maintain gene expression due to transcriptional or epigenetic constraints (e.g. lack of key transcription factors and co-regulators or suppressive chromatin modifications). The degree to which post-transcriptional blocks to HIV-1 gene expression enforce a state of latency in vivo is not yet completely understood, but is under active investigation.

While many forms of persistent proviral DNA may impact the health of infected individuals, only replication-competent proviruses capable of viral rebound after ART cessation are a barrier to curing HIV-1 infection [3, 6]. Collectively, we term the cells that harbor persistent, replication-competent HIV-1 as ***HIV*-*1 reservoir(s)***. While several cell populations have been proposed as HIV-1 reservoirs, the only population yet convincingly shown to be a long-term reservoir for HIV-1 are resting memory CD4^+^ T cells harboring latent but replication-competent HIV-1. We term these cells the ***latent reservoir***. In Fig. 1, we provide a decision tree describing the classification of HIV-1 DNA found in HIV-1 + individuals on suppressive ART, along with a complementary table indicating which forms of persistent HIV-1 DNA are detected by common assays in the field.

## The quantitative viral outgrowth assay (QVOA) defines the latent reservoir

Early studies defining the tropism of HIV-1 indicated that activated CD4^+^ T lymphoblasts were the preferred target of infection. Activated CD4^+^ T lymphoblasts are short-lived effector cells of the immune system, and landmark viral dynamics studies indicated that most productively infected cells displayed a short half-life [27–32]. These initial observations, however, did not reveal that non-replicating forms of infectious HIV-1 might persist indefinitely in infected individuals.

Nonetheless, experimental observations early in the HIV/AIDS epidemic were consistent with some form of HIV-1 persistence. A number of studies indicated that the fraction of mononuclear cells which contained proviral DNA far exceeded those that were positive for HIV-1 RNA [33], suggesting that a subset of apparently infected cells were not transcribing HIV-1 RNA. The first direct experimental evidence for persistence of full-length, replication-competent proviral DNA came in 1995 [1]. In this study, we demonstrated not only that latent integrated proviral DNA could be readily detected in resting CD4^+^ T cells from viremic donors, but that these quiescent proviruses could be induced by activation to produce infectious virus [1]. Definitive identification of the latent reservoir came shortly thereafter. Studies by our group, the Richman group, and the Fauci group demonstrated that replication competent HIV-1 could be recovered from infected individuals on suppressive ART [3–5]. Follow-up longitudinal studies indicated that despite effective ART, the latent reservoir did not appreciably decay (population half-life of 44 months) [6, 8, 34]. This remarkable stability cemented the latent reservoir as the primary barrier to curing HIV-1 infection.

Critical to these early discoveries was the method developed to quantify the frequency of resting CD4^+^ T cells harboring latent HIV-1: the quantitative viral outgrowth assay (QVOA) [3]. In this assay, resting CD4^+^ T cells are purified from the blood of HIV+ donors on suppressive ART and then plated in a limiting dilution format. Because these resting cells do not produce virus without T cell activation [1], any virus production induced in this population reflects the presence of latent HIV-1. To reverse latency and induce virus production, limiting dilutions of these cells are stimulated with a T cell activator (e.g. the mitogen phytohemagglutinin with irradiated allogeneic PBMCs or co-stimulatory antibodies). Released virus is propagated by addition of cells susceptible to infection, allowing exponential expansion of virus produced from a single latently infected cell to the point where detection by viral antigen ELISA is possible. Given the labor and resource intensive nature of the original QVOA protocol, several modifications to the QVOA have been made aiming at improving assay throughput (discussed below). Regardless, the QVOA remains a gold-standard assay for quantifying the latent reservoir.

## Modifications of the QVOA to increase throughput

In essence, successful performance of the QVOA requires complete and reproducible activation of input CD4^+^ T cells to reverse HIV-1 latency, facilitation of outgrowth of reactivated replication-competent proviruses, and detection of that outgrowth. Various modifications of each of these steps have been successfully implemented.

In the original studies, the QVOA was performed on resting memory CD4^+^ T cells to demonstrate the existence of quiescent but replication-competent proviruses. These resting memory CD4^+^ T cells were defined by the absence of accepted cell-surface markers of T cell activation (CD69, CD25, HLA-DR). However, given that the latent reservoir in resting CD4^+^ T cells has now been established as a mechanism of HIV-1 persistence, the purification of resting memory CD4^+^ T cells is not required. In fact, in some instances the purification of resting memory CD4^+^ T cells for QVOA analysis may not be advisable (e.g. after administration of a therapeutic that alters the expression of an accepted cell-surface marker of T cell activation). The QVOA is now routinely performed on bulk CD4^+^ T cells from HIV-1+ donors. In this situation, some infected CD4^+^ T lymphoblasts may also be present. Most of these cells have a short half-life, but in patients on ART they are likely derived from in vivo activation of infected resting CD4^+^ T cells.

The standard QVOA protocol [3, 35, 36] calls for activation of input CD4^+^ T cells via stimulation with the mitogen PHA and allogeneic, irradiated feeder PBMCs. These conditions were selected on the basis of previous reports that they induced activation of roughly 100% of resting T cells [37]. However, alternative activation conditions have been successfully employed in inducing outgrowth of replication-competent HIV-1, including the use of immobilized CD3 and CD28 costimulatory antibodies [4], CD3 and CD28 antibody coated beads [38], and phorbol 12-myristate 13-acetate (PMA) with ionomycin [18]. A large-scale formal comparison of these activation approaches in the QVOA has not yet been completed. However, a recent study did compare the efficacy of these activation approaches in inducing viral outgrowth in the QVOA using samples from five HIV-1 infected donors on suppressive ART, and no significant difference in the induction of viral outgrowth was observed between CD3/CD28 costimulation, PMA and ionomycin, and PHA with allogeneic, irradiated feeders [39]. This aligns with a previous study which showed comparable induction of viral outgrowth by CD3/CD28 antibody coated beads and PHA with allogeneic, irradiated feeders [38].

After successful reactivation of latent proviruses via T cell stimulation, released virus is propagated by the addition of cells susceptible to HIV-1 infection. This step is critical, as the exponential outgrowth of reactivated proviruses definitively establishes these proviruses as replication-competent. While PHA-stimulated, CD8-depleted PBMCs obtained from uninfected donors have traditionally been used to promote viral outgrowth, alternatives have been described. These include the use of bulk PBMCs from uninfected donors [39] and the use of cell lines that support HIV-1 replication (e.g. MOLT-4/CCR5 cells, Sup-T1 cells) [40, 41]. In principle, any cell type that robustly supports HIV-1 replication should be suitable for use in the QVOA [41]. However, cell lines offer many obvious advantages over freshly isolated cells from uninfected donors, namely consistency and significantly reduced resource burden.

Replication-competent proviruses must then be detected to determine the frequency of latent HIV-1 infection. Traditionally, the QVOA is performed in a limiting dilution format and viral outgrowth is detected via p24 antigen ELISA, with the frequency of latent infection calculated based on which well dilutions are positive for outgrowth using limiting dilution [42]. Using this approach, the QVOA requires 14–21 days, largely due to the relative insensitivity of the p24 ELISA. While time intensive, this approach does ensure that only replication-competent viruses are detected.

To reduce the time required to complete the QVOA, alternative methods for detecting viral outgrowth have been described. RT-qPCR detection of HIV-1 RNA in culture supernatant at assay day 7 was shown to be comparable to p24 antigen ELISA at day 14 at identifying outgrowth-positive wells [41], significantly shortening the QVOA. An important caveat to the use of more sensitive detection methods is that some defective proviruses can produce viral RNA and viral protein [24], and therefore it is essential to demonstrate exponential increases over time in the measured viral parameter [41]. A deep sequencing based QVOA was developed in which viral outgrowth was detected and latently infected cell frequencies were estimated via paired-end short-read sequencing of supernatant from bulk cultures, potentially shortening the assay and eliminating the need for limiting dilution [43]. The number of reactivated viruses captured in this bulk culture assay is derived from the number of distinct viral sequences captured. As such, this approach is not capable of distinguishing identical clones of replication-competent virus [14, 17], which may grow out simultaneously in a given bulk culture well. Finally, two recent studies describe the use of the TZM-bl reporter cell line to detect proviruses capable of new integration events [44, 45].

The QVOA enabled the identification of the latent reservoir in resting memory CD4^+^ T cells, but the assay has also been modified to enable the interrogation of latent HIV-1 in other cell types. A viral outgrowth assay for latent SIV in resting macaque CD4^+^ T cells has been previously reported [46, 47], and a recent study describes the adaptation of the QVOA for detection of latent SIV proviruses in myeloid cells [48]. While the mechanisms for inducing the reactivation of latent proviruses in various cell types which may harbor latent proviruses could vary, the general approach of the standard QVOA can be adapted for other cell types to enable the discovery of potential new reservoirs for latent HIV-1.

While the QVOA provides definitive evidence of the persistence of latent HIV-1, recent studies demonstrate that the assay does not capture all replication-competent proviruses in the latent reservoir. Two recent studies by our group demonstrate that additional viral outgrowth can be induced with multiple rounds of T cell activation [10, 17]. In these studies, CD4^+^ T cells from HIV-1 infected donors on ART are subjected to multiple rounds of T cell activation and virus expansion. In most cases, restimulation of cells from wells negative for outgrowth in the initial round of T cell activation increased the total number of latent proviruses detected by the QVOA. These findings demonstrate that not all replication-competent proviruses are reactivated by a given round of T cell activation, and suggest that cells carrying replication-competent HIV-1 can be activated and proliferate without producing virus [10, 17, 49]. Interestingly, identical replication-competent proviruses were obtained from distinct culture wells after different numbers of cycles of T cell activation, suggesting that clonally expanded CD4^+^ T cells harboring replication-competent proviruses may not respond uniformly to a given round of T cell activation. These findings are consistent with other recent reports in vivo [50] and ex vivo [14]. The finding that cells infected with replication-competent virus can be activated and proliferate without producing virus supports the hypothesis that the latent reservoir is maintained in part by cellular proliferation [11, 17, 49–52]. This has important implications for our understanding of the dynamics of the latent reservoir. The fact that the size of the latent reservoir remains relatively stable over time implies equilibrium between the proliferation and clearance of cells comprising the latent reservoir.

Multiple-stimulation QVOA studies have demonstrated that latently infected CD4^+^ T cells are present at a higher frequency than detected by the standard QVOA. The standard QVOA can thus be considered a definitive minimal estimate for the size of the latent reservoir, capturing only a fraction of the latent reservoir with a single round of stimulation and outgrowth. Multiple-stimulation QVOA captures an ever-increasing fraction of the latent reservoir with each successive round of T cell activation and outgrowth. It is conceivable that after a sufficient number of rounds of T cell activation and outgrowth, the multiple-stimulation QVOA would capture all latently infected cells, but this is not experimentally feasible. Critically, these findings indicate that the latent reservoir is not well defined by the proviruses that can be reactivated and captured in the standard or multiple-stimulation QVOA. Rather, the latent reservoir may be better defined as those proviruses that are genetically intact and thus capable of replication in vivo. Under this definition, the latent reservoir can be best interrogated using molecular approaches that distinguish genetically intact proviruses.

## Sequencing-based measures of the latent reservoir

In infected individuals on ART, 300/10^6^ resting CD4^+^ T cells contain HIV-1 proviruses, but only a fraction of these proviruses are replication-competent HIV-1 [9]. The frequencies measured in the QVOA are in the range of 0.1–10 infections units per million (IUPM) resting CD4^+^ T cells. This large difference fueled interest in better understanding the proviral landscape. Several methods have been developed to capture the sequence of individual proviruses to distinguish defective proviruses from those intact ones that are potentially capable of replication.

The importance of such sequencing based approaches is underscored by the surprising results of two recent studies that used a novel and unbiased single genome amplification method to define the sequence landscape of persistent proviruses and examine the nature of defects present in replication incompetent proviruses [10, 13]. In this method, near full-length PCR is performed to amplify proviruses in patient samples [53]. To enable single proviral sequencing, the outer PCR reaction is performed at limiting dilution. This prevents PCR recombination, ambiguous base calls, or competition between short and long templates, and instead allows for the amplification of individual proviruses. Following the outer PCR, inner PCRs are performed to confirm the clonality of the template, followed by a series of overlapping inner PCRs to amplify the complete provirus. The PCR products are run on agarose gels and cut out for direct sequencing to minimize PCR-induced error. Importantly, the amplification conditions must allow efficient detection of individual, full-length proviruses.

Using this initial single proviral-genome sequencing method, we examined the sequences of the proviruses present in QVOA wells that were negative for viral outgrowth [10]. The vast majority of these proviruses were defective, harboring either large internal deletions and/or APOBEC3G-mediated hypermutation. Unexpectedly, however, an appreciable number of non-induced proviruses were intact, and subsequent in vitro infection experiments using base-for-base synthetic reconstructions of these proviruses confirmed their replication competence. These data suggested that the traditional QVOA was not capturing all replication-competent proviruses, but rather only the fraction induced in a single round of T cell activation and outgrowth. This was confirmed by multiple-stimulation QVOA experiments described above [17].

A follow-up study employing the same method interrogated the proviral genomes from HIV-1 infected individuals present in unstimulated resting CD4^+^ T cells taken directly from infected individuals on ART. In this study, the composition of the proviral landscape was compared between viremic individuals and individuals who initiated ART during either acute or chronic infection [13]. Even in subjects treated during the acute/early phase of infection, only a very small fraction (5%) of proviruses are intact. Many of the proviral genomes in these individuals are hypermutated and a smaller fraction have deletions. The same method was applied to proviral DNA from individuals who started treatment during the chronic phase of infection. In this case, most proviruses (98%) were also defective, but the most common defects were internal deletions (80%). Importantly, the median frequency of cells carrying intact proviruses is more than 60-fold higher than what is detected by the standard QVOA. These studies are redefining our understanding of what comprises the latent reservoir, favoring a definition that includes all cells harboring a genetically intact and replication-competent provirus.

The relevance of defective HIV-1 proviruses to the development and assessment of cure strategies is currently being investigated. A recent study demonstrated that hypermutated proviruses and proviruses that have defects in the major splice donor site and/or the packaging signal can be transcribed and that the resulting RNAs can spliced and translated [24]. Additional in vitro analysis indicates that cells carrying these defective proviruses can be recognized and targeted by HIV-1 specific cytotoxic T lymphocytes (CTLs) [41]. These findings support the hypothesis that defective proviruses may produce HIV-1 antigens and perturb adaptive immunity in vivo, potentially contributing to the persistent immune activation observed in patients on ART. Follow-up studies will certainly discern the degree to which these defective proviruses might impact patient health or effect efforts to eliminate the latent reservoir.

Full-length proviral sequencing provides many advantages compared to other methods that probe sub-genomic regions. As discussed above, the proviral landscape is extremely heterogeneous due to HIV-1 intra-host diversity, internal deletions, and deamination-induced hypermutation. As a consequence, all sub-genomic amplification strategies introduce bias, resulting in sequence data that may not be representative of the actual population of HIV-1 proviruses present in a given sample. In particular, sub-genomic PCR studies can vastly overestimate the frequency of latently infected cells by detecting proviruses with lethal defects outside of the region amplified. They also underestimate the total number of infected cells because they fail to amplify proviruses with deletions overlapping the region amplified. Some sub-genomic sequencing assays can overestimate the fraction of identical sequences resulting from clonal expansion, especially in samples with reduced HIV-1 diversity, such as culture supernatants from QVOA or cells from patients treated during the early stages of acute infection [54].

On the other hand, full-length sequencing is time consuming and labor intensive, often requiring custom design of additional primers and multiple sequencing reactions. Two recent studies applied Illumina-based next generation sequencing (NGS) to full-length HIV-1 proviral sequencing [15, 16]. Briefly, end-point diluted proviral DNA is amplified with a nested PCR spanning around 9 kb of the HIV-1 genome. PCR products of variable size are purified and, following library preparation, loaded into the Illumina MiSeq platform. The contigs of single proviruses are constructed by *de*-*novo* assembly mapping reads against a reference genome. The output proviral sequences are then sorted based on the presence of defects that would preclude replication. This new approach is cost-effective and it simplifies the sequencing of difficult regions compared to Sanger approaches while yielding enough sequencing data to study the composition of the proviral landscape in CD4^+^ T cell subsets of interest. The study from Lee and colleagues applied this new sequencing approach to polarized phenotypic subsets of cells sorted based on intracellular cytokine staining [16]. Interestingly, they observed a higher proportion of intact proviruses in Th1 CD4^+^ T cells, which predominate in ART-treated subjects. Recently, Hiener et al. used the FLIP assay (full-length sequencing of individual proviruses) to investigate the composition of proviral DNA in CD4^+^ T cells across the differentiation subsets (Naïve → Central Memory → Transitional Memory → Effector Memory) [15]. This assay identified expanded clones in all subjects (n = 6), for both defective and intact proviruses, supporting the role of cell proliferation in HIV-1 persistence. In addition, the contribution in intact proviruses was uneven across subsets, with effector memory CD4^+^ T cells showing the highest levels of intact genomes, contributing to a median of 75% of all intact proviruses. Surprisingly, central memory CD4^+^ T cells had the lowest levels of intact proviruses. Follow-up studies may be needed to investigate whether sub-optimal sampling and margination of central memory CD4^+^ T cells in secondary lymphoid tissues could have affected these observations.

The studies from Lee et al. and Hiener et al. pioneered the use of NGS to characterize HIV-1 persistence and contributed to our understanding of the complexity of the proviral landscape. However, the composition of the HIV-1 reservoir is extremely variable, and it can be difficult to generalize the findings observed in a limited number of subjects in a single time point. In addition, the abundance and species richness of HIV-1 infected clones is still unpredictable and a small number of highly expanded clones can skew the global picture within each patient at a given time point.

## Unmet needs in measuring the latent reservoir

The development of assays that measure HIV-1 persistence has been invaluable in furthering our understanding of the impact of ART on viral replication and the nature of the latent reservoir. However, current methods have several limitations and the quantification of the latent reservoir remains challenging.

Limit of detection and dynamic range are two of the biggest constraints of current assays, especially in the context of interventions based on purging of the reservoir or on very early treatment. Hematopoietic stem cell transplantation (HSCT) can lead to dramatic reductions in the size of the latent reservoir (up to 4 logs) [55–57], making the detection of residual infected cells nearly impossible with the available sampling capacity, both in peripheral blood and in lymphoid tissues. Similar challenges are present when ART is initiated during the earliest phases of infection (eclipse to Fiebig I phases), as recently reported in two cases of hyper-acute infection diagnosed in the setting of PrEP trials [58]. Despite extensive testing with available assays, persistent virus may not be detected in such patients simply because no infected cells are present in the samples analyzed. The only practical approach is to intensively monitor viral rebound upon ART interruption. The duration of remission in the absence of ART and the rebound kinetics have been extremely informative. For example, mathematical models based on viral dynamics can relate the degree of reservoir reduction to the time to rebound after ART interruption. The models have been used to estimate both the efficacy of latent reservoir reduction strategies and the number of residual infected cells responsible for the rebound [59, 60]. In the cases of the Boston patients and the PrEP patient described by Henrich et al., such models predicted that only a few hundred cells harboring latent, replication competent HIV-1 were present in the patients at the time of ART interruption.

These difficulties can be extrapolated to larger clinical trials involving new molecules aimed at reducing the latent reservoir, such as latency reversing agents (LRA), therapeutic vaccines, CAR T cells, and/or ADCC-competent broadly neutralizing anti-HIV-1 antibodies. In such trials, both the magnitude of changes in reservoir size and the scale of response to a given treatment might vary across study participants, requiring large sample sizes to demonstrate efficacy. In this context, the assay(s) used to quantify HIV-1 persistence would need adequate throughput to process hundreds of samples and be capable of reliably detecting even modest changes in reservoir size. As discussed above, approaches that allow the quantification of replication-competent proviruses are labor and time consuming, which makes them impractical for large cohort studies. PCR-based assays that measure HIV-1 DNA have a higher throughput, but cannot distinguish replication competent from defective proviruses. Infected cells carrying intact versus defective proviruses may respond very differently to a given intervention, and total HIV-1 DNA data could be difficult to interpret or even misleading, given the “background noise” of defective proviruses.

Continuous efforts are needed to develop new approaches that can measure and characterize that true “minimal residual disease” that must be targeted by future curative strategies. We propose that the next generation of assays designed to measure the HIV-1 reservoir should have a low and “modular” limit of detection, a wide dynamic range, the capacity to distinguish intact proviruses, and should be scalable for studies with large samples sizes. As the need for such assays in clinical trials grows, rigorous studies to qualify or validate such assays will be important, and the clinical utility of any given assay will need to be formally established. It is our view that efforts to design and implement new assays for the HIV-1 latent reservoir will ultimately help to enable the discovery and development of new therapeutics aimed at curing the infection.
